# Pro-equity legislation, health policy and utilisation of sexual and reproductive health services by vulnerable populations in sub-Saharan Africa: a systematic review

**DOI:** 10.1177/1757975920941435

**Published:** 2020-08-04

**Authors:** Muriel Mac-Seing, Kate Zinszer, Charity Oga Omenka, Pierre de Beaudrap, Fereshteh Mehrabi, Christina Zarowsky

**Affiliations:** 1 Department of Social and Preventive Medicine, School of Public Health, Université de Montréal, Montreal, Canada; 2 Centre de recherche en santé publique, Université de Montréal et CIUSSS du Centre-Sud-de-l’Île-de-Montréal, Montreal, Canada; 3 Centre Population et Développement (CEPED), Institut de recherche pour le développement, Paris, France; 4 Department of Health Management, Evaluation and Health Policy, School of Public Health, Université de Montréal, Montreal, Canada

**Keywords:** determinants of health, equity/social justice, maternal health, policy/politics, reproductive health, sub-Saharan Africa, systematic review

## Abstract

Twenty-five years ago, the International Conference on Population and Development highlighted the need to address sexual and reproductive health (SRH) rights on a global scale. The sub-Saharan Africa region continues to have the highest levels of maternal mortality and HIV, primarily affecting the most vulnerable populations. Recognising the critical role of policy in understanding population health, we conducted a systematic review of original primary research which examined the relationships between equity-focused legislation and policy and the utilisation of SRH services by vulnerable populations in sub-Saharan Africa. We searched nine bibliographic databases for relevant articles published between 1994 and 2019. Thirty-two studies, conducted in 14 sub-Saharan African countries, met the inclusion criteria. They focused on maternal health service utilisation, either through specific fee reduction/removal policies, or through healthcare reforms and insurance schemes to increase SRH service utilisation. Findings across most of the studies showed that health-related legislation and policy promoted an increase in service utilisation, over time, especially for antenatal care, skilled birth attendance and facility-based delivery. However, social health inequalities persisted among subgroups of women. Neither the reviewed studies nor the policies specifically addressed youth, people living with HIV and people with disabilities. In the era of the sustainable development goals, addressing health inequities in the context of social determinants of health becomes unavoidable. Systematic and rigorous quantitative and qualitative research, including longitudinal policy evaluation, is required to understand the complex relationships between policy addressing upstream social determinants of health and health service utilisation.

## Introduction

At the 1994 International Conference on Population and Development (ICDP), the international community adopted the Programme of Action (PoA) which recognised sexual and reproductive health (SRH) as a fundamental right (1). This commitment was further renewed during the 2019 Nairobi Summit (2). Building on the Millennium Development Goal (MDG) 5 which focused on improving maternal health (2000–2015), the health-focused Goal 3 of the Sustainable Development Goals (SDG) (2015–2030) reaffirms the importance of ‘universal access to sexual and reproductive health services, including [. . .] family planning, information and education, and the integration of reproductive health into national strategies and programmes (3)’. The SDG agenda on SRH rights (SRHR) catalyses both SDG 3 on health and SDG 5 on gender equality, beyond the MDG 5 objectives (3). Typically, SRHR not only focus on information and services related to contraception, maternal health and HIV/AIDS, but also on the sexual health of adolescents, abortion and gender-based violence (4). Despite notable improvements in several health outcomes from maternal mortality to HIV survival globally, the sub-Saharan African region did not see the same magnitude of change in these indicators. Compared to other regions worldwide, sub-Saharan Africa had the highest average maternal mortality ratio in 2017 (5) and HIV prevalence in 2018 (6).

Social determinants of health such as gender, wealth, and place of residence are reported to influence the accessibility of SRH services, while evidence has shown that structural determinants such as laws and policies, driven by socio-cultural values, can both promote SRHR and restrict the use of specific SRH services such as safe abortion (4). The Commission on Social Determinants of Health (CSDH) report (7) reminds us that social health inequities result from unjust distribution of power and resources as well as inadequate social policies which can worsen people’s health (8), most affecting vulnerable populations. Despite the challenges of defining vulnerability and how best to measure it (9), there is an agreement that vulnerable populations share a complex confluence of common characteristics based on factors such as age, sex, ethnicity, education and wealth, which put them at a heightened disadvantage relative to other populations (10). One of the key CSDH recommendations was the promotion of a systematic contextual analysis of health disparities among populations (7). Policy approaches to reducing health inequities have been identified, such as ‘targeting disadvantaged populations, closing the gaps between worse-off and better-off groups, and addressing the social health gradient across the whole population’ (11). Considering the above conceptual and methodological context, we were interested in learning more about how the empirical literature addresses the interplay between legislation and policy adoption aimed at reducing health disparities between groups and health service utilisation among vulnerable populations. This paper reports a systematic review which aimed at examining the relationships between health equity-focused legislation and policy, and the utilisation of SRH services by vulnerable populations in sub-Saharan Africa.

## Methods

We followed the structure of the Preferred Reporting Items for Systematic Reviews and Meta-Analyses Statement and used the PICO methodology: Population, Intervention, Comparator (when available), and Outcome (12) (Checklist 1-Supplementary material). The systematic review protocol is registered in the PROSPERO database (https://www.crd.york.ac.uk/PROSPERO/display_record.php?RecordID=106876&VersionID=1184126). We searched the following nine bibliographic databases: CINAHL, EBM Cochrane Systematic Reviews, Embase, Global Health, MEDLINE, Popline, Proquest Dissertations and Theses Global, Scopus and Web of Science. Search terms were developed based on the key concepts related to the research objective: 1) equity, defined as ‘the absence of avoidable or remediable differences among groups of people, whether those groups are defined socially, economically, or geographically’ (13); 2) legislation, defined as any preparation and enactment of laws (14) and/or health policy, defined as any ‘decisions, plans, and actions that are undertaken to achieve specific health care goals within a society’ (15); 3) SRH service utilisation (1) referred to as antenatal care, facility-based delivery, contraception, safe abortion and prevention of mother to child transmission of HIV (PMTCT); 4) vulnerable populations, defined as ‘groups who, because of their position in the social strata, are commonly exposed to contextual conditions that distinguish them from the rest of the population’ (10) such as women, youth and the poor; and 5) countries in sub-Saharan Africa (Supplemental Figure 1). Inclusion criteria were original primary qualitative, quantitative and mixed methods studies which addressed the above research concepts, conducted and published between 1994 (year of the ICPD) and 2019 in sub-Saharan Africa, from both English and French peer-reviewed and grey literature. Search records were independently screened by authors (MMS, COO and KZ).

One author (MMS) extracted data from included studies and another (FM) reviewed them as per the following information: publication year, authors, countries in sub-Saharan Africa, research methods and design, type of legislation/policy adoption/implementation, population and number, type of SRH service utilisation, quantitative and/or qualitative results in SRH service use, and number of years before/after legislation/policy adoption. We analysed the study findings as per the type of legislation/policy which promoted SRH service utilisation per year and country; groups of the population that can be in situations of vulnerability; direction and significance of the SRH results in quantitative research designs, such as quasi-experimental designs which warrant analysis related to causal inferences (16); and quality of reporting in studies. Due to heterogeneity in study outcomes and findings, a meta-analysis was not considered. Rather, we conducted a narrative synthesis (17).

Two authors (MMS and FM) assessed the quality of studies through quality appraisal tools for different study designs, and a third author (KZ) spot checked. The Joanna Briggs Institute’s Checklist for Quasi-Experimental Studies (18) was used to assess the quality of four types of quasi-experimental designs: Category A – without control groups, Category B – with control groups but without pretests, Category C – with control groups and pretests, and Category D – interrupted time-series (19). For cross-sectional studies, the Strengthening the Reporting of Observational Studies in Epidemiology-Combined tool was used (20). For mixed methods studies, the Mixed Methods Appraisal Tool was selected (21). Given the recommendations of the Cochrane Collaboration’s tool for assessing risk of bias, quality scores were not used as they are not deemed appropriate (22). Since no primary qualitative studies were included in the review, no checklist assessing the rigour of qualitative studies was used.

## Results

The initial search produced a total of 5414 references. Of those, 818 duplicates were removed. We then reviewed 4596 references of which 4538 references were discarded based on the inclusion criteria. At the eligibility phase, 58 studies were fully reviewed, of which 32 were finally included (Supplemental Figure 2), involving 14 countries in sub-Saharan Africa where the effects of adopted equity-focused SRH-related legislation and policy were examined. Ghana (*n* = 11) was the country mostly studied, followed by Kenya (*n* = 5), Burkina Faso (*n* = 4) and Mali (*n* = 4). Most studies focused on maternal health service utilisation, and a few examined abortion services, PMTCT, and postnatal care (Supplemental Figure 3). Of these 32 studies, 30 adopted quantitative designs and two studies employed mixed methods. No primary qualitative studies were included in the final phase as they did not meet the combination of inclusion criteria. Among the quantitative studies, there were 26 quasi-experimental studies, with the following study design categories: 11 were in Category A (without control groups) (23–33), four in Category B (with control groups but without pretests) (34–37), eight in Category C (with control groups and pretests) (38–45), and three in Category D (interrupted time series) (46–48). Four studies were cross-sectional (49–52). Among the two mixed methods study designs, one used a quasi-experimental of Category C design along with key informant interviews (53), and the second used a cross-sectional design combined with qualitative interviews (54). Supplemental Table 1 summarises the study characteristics.

### Type of legislation and SRH services used

Among included studies, national legislation or policy adoption promoting the access to and utilisation of SRH services spanned the period from 1996 to 2013, with a concentration of studies conducted between 2000 and 2009 (Supplemental Figure 4). Most studies analysed SRH service utilisation from one to eight years before and one to eight years after legislation/policy adoption (23,25,27,30,31,33,38,42–48) and between 1 and 14 years after legislation/policy adoption (26,28,29,32,34,37,40,41,49–54). Two studies examined service utilisation two to four years before legislation/policy adoption at time point 1 and the same year at time point 2 (24,39). Two others assessed service use the same year as legislation/policy adoption (35,36). Twenty studies out of 32 examined maternal health-related policies which focused on eliminating or subsidising facility-based delivery (23,24,29–31,38,41,42,44,45,48,51,53) and skilled birth attendant use (24,27–30), either through specific policies promoting these services or through national health care reforms (25), national health insurance schemes (27,37,46,54), and performance-based financing (40). Fourteen studies examined the effects on antenatal care service utilisation from the influence of maternal health fee exemptions or abolition (29,30,32,43,48,51,52), performance-based financing (40), specific reproductive health voucher programmes (35,44) and health insurance schemes (26,36,37,54). Two studies looked at the impact of abortion legislation on the use of safe abortion service (49) and contraception (50). Four other studies examined the effects of a reproductive health programme (35), performance-based financing (40), national health insurance (32), and exemption fees (32) or free health care (29) for pregnant women and lactating mothers on family planning and contraception. Five studies considered policy pertaining to caesarean sections (33,34,45,47,53). To a lesser extent, postnatal care (35,43,44,52) and PMTCT (29,39) were studied.

### Changes in SRH service utilisation

Most studies (27/32) used the four types of quasi-experimental designs. They examined a large range of multiple SRH outcomes (*n* = 46), and their findings varied in significance (Supplemental Table 2). Sixty percent (28/46) of the results found statistically significant positive increases following policy implementation in service utilisation, including family planning and contraception (32,35,40), antenatal care (32,36,37), facility-based delivery (23,24,29,35,37,38,40,43,45,46,53), skilled birth attendant use (24,27–29), caesarean section (32,34,45,47,53), postnatal care (44) and PMTCT (39). Among these studies with positive results (statistically significant and improved outcomes), several examined the effects of abolition/reduction of service fees (23,29,32–34,38,43,45–47,53) and the implementation of national health insurance schemes (32,36,37). Eight studies found mixed results (i.e. a mixed of positive, negative, statistically significant and not statistically significant outcomes) on the use of antenatal care (40,43), facility-based delivery (26,42,44,48), skilled birth attendant use (25) and caesarean section (33). Ten others showed no significant results on the use of antenatal care (26,30,35,48), facility-based delivery (30,31,41,53), skilled birth attendants (30) and HIV testing during pregnancy (29). From all quasi-experimental study designs, no specific reporting on safe abortion care service utilisation was made.

### Differential vulnerability in the utilisation of SRH services among population sub-groups

Despite the general trend of increased utilisation of SRH services following the adoption of legislation or health policy, disparities in service utilisation remained among sub-groups of women or vulnerable women. Women with no education and within the lowest wealth quintile were less likely to use antenatal care in Ghana even after fee exemption (52). Concerning the uptake of facility-based delivery, women who had some education (38), high school or higher education (24), those who were wealthier (24,35,40,41,46), residing less than 5 km away from a health facility (51) or living in less difficult terrain (29) tended to give birth in health facilities more often than the other groups of women. Related to caesarean section use, in some studies, less disadvantaged women benefited more from services (34,47,53), while one study in West Africa showed that non-educated women and those living in rural areas benefited most after policy adoption (45). A study conducted in Kenya found that women who were of Muslim/Other/No religion were more inclined to use family planning compared to women of Catholic faith (35). Besides sub-groups of women of reproductive age studied, a South African study examined the utilisation of contraception among adolescent mothers (50) and a Uganda study focused on HIV testing among pregnant women and their male partners (39). Included studies did not address other vulnerable populations as their primary targets or in explicit sub-analyses.

### Quality of reporting in studies

Among the two mixed methods studies, only one of the five quality assessment criteria was addressed, which was related to the rationale for why a mixed methods design was important. Despite having reported the use of qualitative data collection techniques, both studies heavily focused on their quantitative results and interpretation. The other criteria on mixed quantitative and qualitative methods data integration, interpretation and management were not reported. Regarding the assessment of 27 quasi-experimental study designs, three main observations emerged. First, in eight studies, mostly using repeated cross-sectional surveys (23,29,30,32,34,38,42,53), there was no indication that the independent variables occurred in time before the dependent variables (18), even though the year of legislation/policy adoption was known in all studies. Second, 14 studies did not include any control groups (23–33,46–48). Third, on a more positive side, six studies added multiple measurements at different time points before and after the intervention (23,39,43,46–48). Concerning the five cross-sectional studies, none clearly reported efforts to address potential sources of bias such as controlling for confounding factors. Further, three studies (49,50,52) out of five did not clearly report how quantitative variables were handled in the analyses or statistically control for confounding factors. All cross-sectional studies acknowledged methodological limitations.

## Discussion

To the best of our knowledge, this is the first systematic review to assess the scientific literature which examined the relationships between legislation or health policy and the utilisation of SRH services by vulnerable populations in sub-Saharan Africa. We found that the adoption of equity-focused legislation and policy promoted SRH service utilisation over time, mainly related to maternal health services among vulnerable populations of women, corroborating what has been reported in the literature (55–57). However, despite the passage of time since the ICPD promoting a wide range of SRH rights and services for all, a narrow scope of SRH focusing on maternal health service utilisation is observed. This may be explained by the emphasis of the MDG 5, from 2000 to 2015, to prevent and manage the ‘clustering of mortality around delivery’, and save women’s lives (58).

### Promising pro-equity policy influence over SRH service utilisation

We found that policies promoting fee abolition or reduction and national health insurance schemes seemed to lead to increased trends in various types of SRH service utilisation and across groups of populations, including those less educated, less better-off and living in rural areas. These policies addressed social determinants such as education, wealth and place of residence across different groups and social gradients in the population (11). Despite promising improvements over time, social health inequities persisted within vulnerable populations based on the rich/poor, educated/non-educated and urban/rural divides. A systematic review of differences in maternal health service utilisation in low and middle income countries (LMIC) showed that living in urban areas and being better off economically positively influenced the use of skilled birth attendants and likelihood of delivering in a health facility, while economic status did not influence antenatal care uptake (59). The age and parity of mothers, as well as a woman’s education and that of her husband’s, have been described in the literature as factors for divergent outcomes in relation to antenatal care uptake (60), while societal norms and values (7), such as religion were reported as potential barriers for family planning use (61). Further, abortion laws remain very restrictive in most of the African continent with only South Africa and Cape Verde legally allowing women to request an abortion, under specific conditions (62). As for the utilisation of PMTCT services, barriers to policy translation into concrete changes could be partly explained by stigma and fear of HIV status disclosure to partners and family.

### Important populations left behind

Studies included in this review excluded specific vulnerable populations. Sub-Saharan Africa is home to three of the world’s largest vulnerable populations, notably youth, people living with HIV and people with disabilities. Firstly, though several studies included various sub-groups of women in their reproductive age, the majority did not report any specific analysis pertaining to young people. Among young women, 37% and 45% are married before they reached 18 years old in Eastern and Southern Africa and Western and Central Africa, respectively (63). Over the past decades, single young women in sub-Saharan Africa have become more sexually active; this has important practical implications for SRH service utilisation by youth (64).

Secondly, the majority of people living with HIV worldwide live in sub-Saharan Africa, with women aged 15 and older representing 59% of new adult HIV infections in 2017 (65). Facing multiple challenges such as stigma and discrimination at family and community levels, the SRHR of people living with HIV are curtailed by laws criminalising the transmission or non-disclosure of HIV transmission, which jeopardise their SRH service utilisation (65). According to development aid assistance analyses from 2000 to 2013, HIV/AIDS has received the majority of external funding relative to other health sectors (66). The high level of foreign assistance to many sub-Saharan African countries where HIV was prevalent may have shrunk the domestic policy space for policy formation because of aid dependence (67).

Thirdly, people with disabilities represent approximately one billion people of the world’s population, and 80% live in LMICs, including in sub-Saharan Africa (68). Literature has shown that people with disabilities experience barriers related to physical and communication accessibility, negative attitudes of health professionals, and financial costs when accessing SRH services (69,70). A systematic review and meta-analysis reported that adults with disabilities in sub-Saharan Africa, especially women, were at heightened risk for HIV (71). A recent study on the intersection between gender, disability, and poverty in Kenya reported that despite pro-poor policy promoting free maternal healthcare, women with disabilities were left behind (72). Although these three large groups stood out by their absence in this review, other vulnerable groups such as people living on the streets and sex workers were also missing. The use of conventional surveys measuring health disparities might not reach them or address their specific characteristics, which may explain this gap (4).

### Limitations of the literature, PICO methodology and the study

This review highlighted limitations in the literature and the use of a classic PICO systematic review methodology to explore complex questions. The study itself also has several limitations. First, despite having adopted a systematic review process covering a 25-year period (1994–2019), our literature search resulted in only 32 studies. This demonstrates that the relationships between pro-equity legislation or health policy and the utilisation of SRH services by people in situations of vulnerability are largely unexplored in sub-Saharan Africa. Second, the positivist nature of the PICO methodology requiring a specific relation between various research question components could have precluded the inclusion of qualitative research studies. The standardised PICO requirements are often in contradiction with the more inductive nature of qualitative research. Third, our choice to review primary empirical research meant that we did not include realist and systematic reviews which may have led to other angles of analysis. Fourth, while most studies focused on the ‘impact’ aspect of the CSDH recommendation to evaluate health policy more effectively (7), none of the included studies looked at the effects of, for example, pro-poor tax policy, gender equality policy, or disability laws on the utilisation of SRH services among vulnerable populations. Finally, the quality assessment of studies suggested methodological weaknesses such as ambiguous temporality between independent and dependent variables in cross-sectional surveys (not related to when a legislation/policy was adopted versus when a study was implemented), selection and history, which potentially threaten the internal validity of studies (19).

## Conclusion and implications for policy and research

In the SDG era with the motto ‘leave no one behind’, policy- and decision-makers need to revisit national legislation and policy implementation more critically and address a broader scope of SRH services beyond maternal health care to reach the SRHR targets of 2030 (4). In terms of policy, not only is it essential to remove financial barriers and reduce SRH service utilisation disparities among groups, but there is also an urgency to consider social determinants of health (7) so as to address the unequal distribution of socioeconomic factors such as income, education and place of residence (11). This calls for more integrated intersectoral action between the health, finance and economy, education and infrastructure sectors, for instance (11,73). The attainment of SRH universal coverage is multifaceted and depends upon the interplay of power structures (e.g. sexism, classism, etc.) which produce and perpetuate unequal health outcomes. An intersectional analysis can make health inequities more visible in relation to these power dynamics (74). Regarding research, this review also confirmed the need for more rigorous quantitative, qualitative and mixed methods research designs to answer to research questions emanating from complex policy and health system related contexts. Specifically, research strategies such as the case study approach, advances in impact evaluation, investigating policy and system change over time, cross-national analysis and action research are suggested for policy analysis and systems strengthening (75). Research should further examine prospectively or retrospectively the impacts of legislation/policy implementation on SRH service utilisation, over a period of at least 10 years (75). In conclusion, health policy and systems research should also be more ‘people-centred’, in particular focusing on the most vulnerable, in developing recommendations for policy- and decision-makers ‘to address equity and social justice’ more systematically (76).

## Supplemental Material

Supp_mat – Supplemental material for Pro-equity legislation, health policy and utilisation of sexual and reproductive health services by vulnerable populations in sub-Saharan Africa: a systematic review Click here for additional data file. Supplemental material, Supp_mat for Pro-equity legislation, health policy and utilisation of sexual and reproductive health services by vulnerable populations in sub-Saharan Africa: a systematic review by Muriel Mac-Seing, Kate Zinszer, Charity Oga Omenka, Pierre de Beaudrap, Fereshteh Mehrabi and Christina Zarowsky in Global Health Promotion
