# Supplementary material for: Pro-equity legislation, health policy and utilisation of sexual and reproductive health services by vulnerable populations in sub-Saharan Africa: a systematic review
Source: Glob Health Promot. 2020 Aug 4;27(4):97–106. doi: 10.1177/1757975920941435 (PMC7750661; doi:10.1177/1757975920941435)
Supplement: Supp_mat – Supplemental material for Pro-equity legislation, health policy and utilisation of sexual and reproductive health services by vulnerable populations in sub-Saharan Africa: a systematic review [file Supp_mat.pdf]

Checklist 1. Preferred Reporting Items for Systematic Reviews and Meta-Analyses (PRISMA)

PRISMA Section/topic	#	Checklist item	Reported on page #
**TITLE**			
Title	1	Identify the report as a systematic review, meta-analysis, or both.	Title page
**ABSTRACT**			
Structured summary	2	Provide a structured summary including, as applicable: background; objectives; data sources; study eligibility criteria, participants, and interventions; study appraisal and synthesis methods; results; limitations; conclusions and implications of key findings; systematic review registration number.	Abstract
**INTRODUCTION**			
Rationale	3	Describe the rationale for the review in the context of what is already known.	1-2
Objectives	4	Provide an explicit statement of questions being addressed with reference to participants, interventions, comparisons, outcomes, and study design (PICOS).	2
**METHODS**			
Protocol and registration	5	Indicate if a review protocol exists, if and where it can be accessed (e.g., Web address), and, if available, provide registration information including registration number.	2
Eligibility criteria	6	Specify study characteristics (e.g., PICOS, length of follow-up) and report characteristics (e.g., years considered, language, publication status) used as criteria for eligibility, giving rationale.	2-3
Information sources	7	Describe all information sources (e.g., databases with dates of coverage, contact with study authors to identify additional studies) in the search and date last searched.	2
Search	8	Present full electronic search strategy for at least one database, including any limits used, such that it could be repeated.	Figure 1
Study selection	9	State the process for selecting studies (i.e., screening, eligibility, included in systematic review, and, if applicable, included in the meta-analysis).	2
Data collection process	10	Describe method of data extraction from reports (e.g., piloted forms, independently, in duplicate) and any processes for obtaining and confirming data from investigators.	3
Data items	11	List and define all variables for which data were sought (e.g., PICOS, funding sources) and any assumptions and simplifications made.	2-3
Risk of bias in individual studies	12	Describe methods used for assessing risk of bias of individual studies (including specification of whether this was done at the study or outcome level), and how this information is to be used in any data synthesis.	3
Summary measures	13	State the principal summary measures (e.g., risk ratio, difference in means).	N/A
Synthesis of results	14	Describe the methods of handling data and combining results of studies, if done, including measures of consistency (e.g., I^2) for each meta-analysis.	3

Section/topic	#	Checklist item	Reported on page #
Risk of bias across studies	15	Specify any assessment of risk of bias that may affect the cumulative evidence (e.g., publication bias, selective reporting within studies).	3
Additional analyses	16	Describe methods of additional analyses (e.g., sensitivity or subgroup analyses, meta-regression), if done, indicating which were pre-specified.	N/A
**RESULTS**			
Study selection	17	Give numbers of studies screened, assessed for eligibility, and included in the review, with reasons for exclusions at each stage, ideally with a flow diagram.	4
Study characteristics	18	For each study, present characteristics for which data were extracted (e.g., study size, PICOS, follow-up period) and provide the citations.	Table 1
Risk of bias within studies	19	Present data on risk of bias of each study and, if available, any outcome level assessment (see item 12).	5-6
Results of individual studies	20	For all outcomes considered (benefits or harms), present, for each study: (a) simple summary data for each intervention group (b) effect estimates and confidence intervals, ideally with a forest plot.	5-6
Synthesis of results	21	Present results of each meta-analysis done, including confidence intervals and measures of consistency.	Narrative
Risk of bias across studies	22	Present results of any assessment of risk of bias across studies (see Item 15).	6-7
Additional analysis	23	Give results of additional analyses, if done (e.g., sensitivity or subgroup analyses, meta-regression [see Item 16]).	N/A
**DISCUSSION**			
Summary of evidence	24	Summarize the main findings including the strength of evidence for each main outcome; consider their relevance to key groups (e.g., healthcare providers, users, and policy makers).	6-9
Limitations	25	Discuss limitations at study and outcome level (e.g., risk of bias), and at review-level (e.g., incomplete retrieval of identified research, reporting bias).	9-10
Conclusions	26	Provide a general interpretation of the results in the context of other evidence, and implications for future research.	10
**FUNDING**			
Funding	27	Describe sources of funding for the systematic review and other support (e.g., supply of data); role of funders for the systematic review.	Online

From: Moher D, Liberati A, Tetzlaff J, Altman DG, The PRISMA Group (2009). Preferred Reporting Items for Systematic Reviews and Meta-Analyses: The PRISMA Statement. PLoS Med 6(6): e1000097. doi:10.1371/journal.pmed1000097 http://www.prisma-statement.org/

Figure 1. Search Strategy

Search components	Search key words
Equity	health equit* OR healthcare equit* OR “health care equit* OR heatlh equalit* OR healthcare equalit* OR health care equalit* OR health inequit* OR healthcare inequit* OR health care inequit* OR health inequality* OR healthcare inequality* OR health care inequality* OR health disparit* OR healthcare disparit* OR health care disparit* OR pro-poor OR pro poor OR socioeconomic status OR socio-economic status OR socioeconomic factor* OR socio-economic factor*
	AND
Legislation/policy	legislation OR law* OR regulation* OR health polic* OR health plan* OR health strateg*
	AND
Sexual and reproductive health service utilisation	utilization of reproductive health OR sexual health OR sexual education OR family planning OR contracept* OR prenatal care OR antenatal care OR facility-based delivery OR facility based delivery OR postnatal care OR postpartum care OR hiv OR aids OR hiv/aids OR hiv testing and counselling OR hiv testing and counseling OR VCT OR sti* OR sexually transmitted infection* OR sexually transmitted disease* OR sexual abuse OR domestic violence OR intimate partner violence OR safe abortion OR post-abortion care OR post abortion care
	AND
Vulnerable populations	vulnerable population* OR vulnerable group* OR special population* OR special group* OR marginalized population* OR marginalized group* OR marginalised population* OR marginalised group* OR underserved population* OR underserved group* OR disadvantaged population* OR disadvantaged group* OR women OR woman OR youth OR adolescen* OR young adult* OR handicap* OR disabilit* OR disabled OR person* with disability* OR people with disability* OR “person* with a disability* OR lgbt* OR transgender OR sexual minorit* OR ethnic minorit* OR indigenous OR homeless OR illiterate OR unemployed OR rural population* OR poor OR street people OR street teen*
	AND
Sub-Saharan Africa	Africa south of the Sahara OR sub-Saharan Africa OR west* Africa OR east* Africa OR central Africa OR south* Africa OR Angola OR Burundi OR Democratic Republic of Congo OR DRC OR Cameroon OR Central African Republic OR CAR OR Chad OR Republic of Congo OR Equatorial Guinea OR Gabon OR Kenya OR Nigeria OR Rwanda OR Sao Tome and Principe OR Tanzania OR Uganda OR Sudan OR South Sudan OR Djibouti OR Eritrea OR Somalia OR Botswana OR Comoros OR Lesotho OR Madagascar OR Malawi OR Mauritius OR Mozambique OR Namibia OR Seychelles OR South Africa OR Swaziland OR Zambia OR Zimbabwe OR Benin OR Mali OR Burkina Faso OR Cape Verde OR Ivory Coast OR Gambia OR Ghana OR Guinea OR Guinea Bissau OR Liberia OR Mauritania OR Niger OR Senegal OR Sierra Leone OR Togo

Figure 2. Flow chart

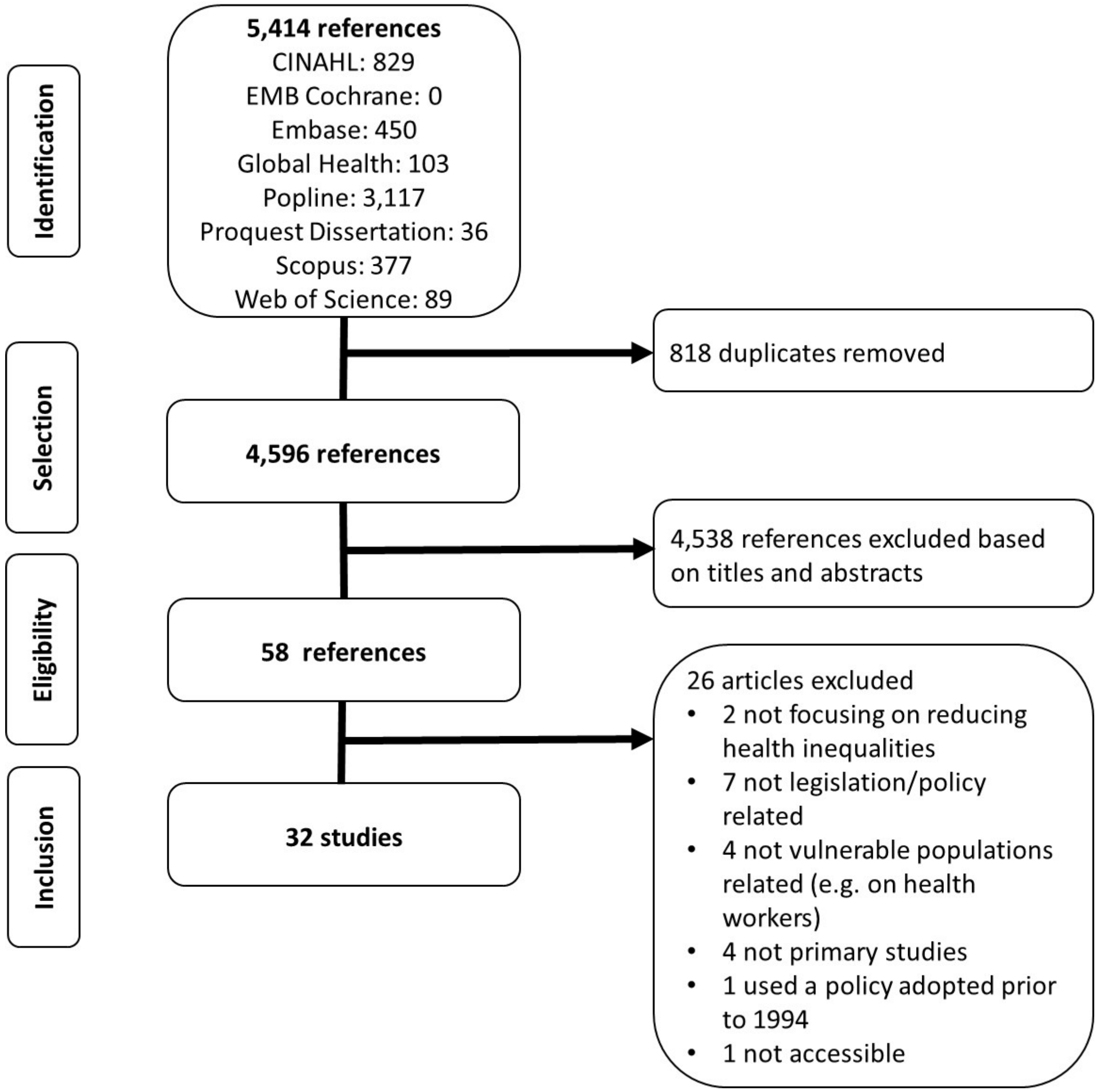

Figure 3. SRH services targeted by legislation/policy per country

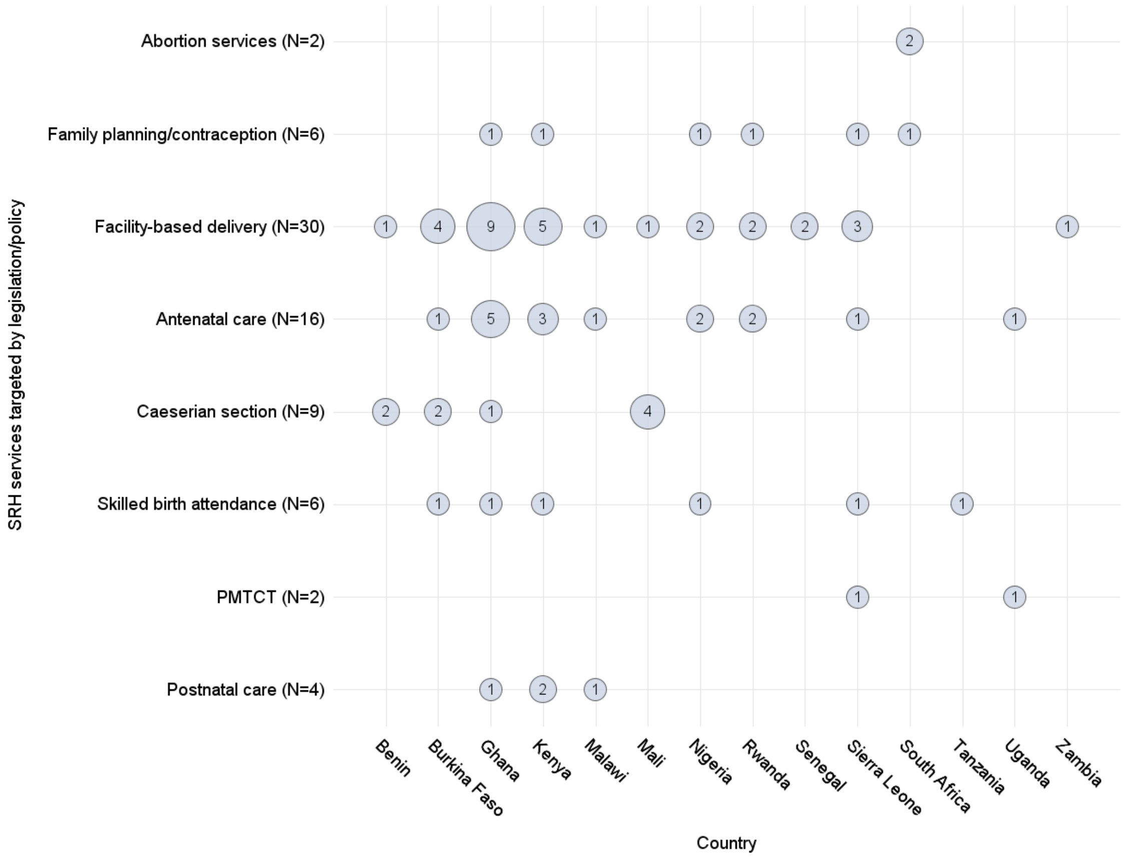

Note:
Studies conducted in a country can look at more than one type of legislation/policy in relation to SRH service utilisation. Hence, one study can examine several countries, policies and the utilisation of SRH services at the same time.

Figure 4. Legislation/policy addressing SRH service utilisation per year

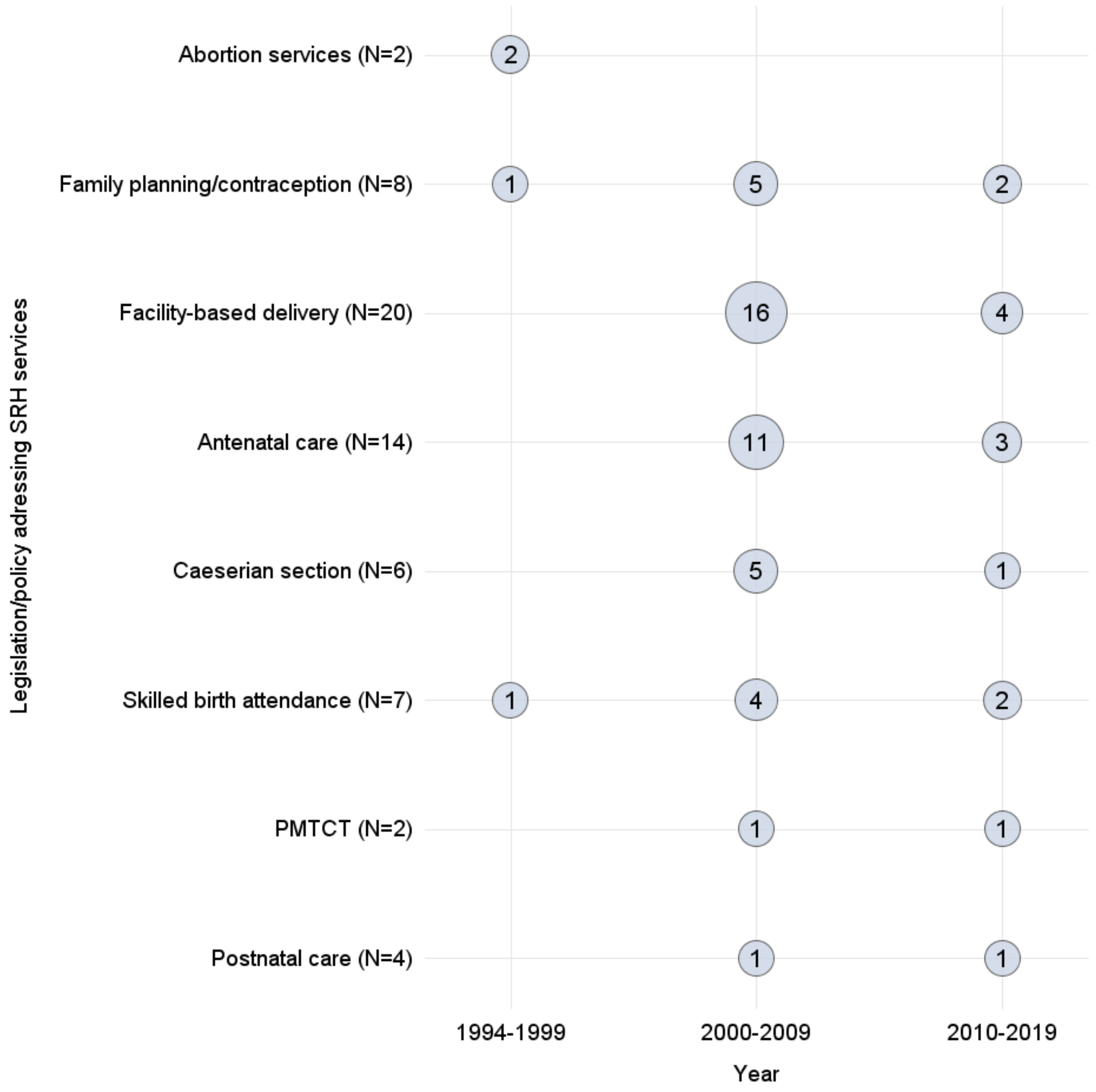

Table 1. Summary of studies included

First author (Year)<br><br>Countries	Research design and methods<br><br>(Period)	Legislation and policy	Type of SRH service utilisation	Population and number	Main results	Difference between the beginning of policy and beginning of study (year)
Dickson (2003)<br><br>South Africa	Cross-sectional<br><br>Survey (September to December 1999)	The Choice on Termination of Pregnancy Act of 1996 – adopted in 1997	1st and 2nd trimester abortion services	Women 16-50 years old from 292 health facilities	▪ 32% of 292 health facilities providing abortion services in country<br>▪ 3,112 (78%) terminations out of a mean of 3,996 abortions/month in the 1st semester<br>▪ 884 (22%) out of a mean of 3,996 abortions/month in 2nd semester<br>▪ 2 urbanised provinces with more access to abortion services	2 years after
Ehlers (2003)<br><br>South Africa	Cross-sectional<br><br>Exploratory descriptive survey (1999-2000)	The Choice on Termination of Pregnancy Act of 1996 – and contraceptives and emergency contraceptives made free	Knowledge and utilisation of contraceptives	250 adolescent mothers aged 19 years old and younger who delivered during	▪ Out of 250 mothers, 139 knew about contraceptives; 117 (46.8%) ever used contraception<br>▪ 94% of 250 used contraceptives after delivery; 65.2% used injections so that family and boyfriends do not need to know about it<br>▪ Only 34 (13.6%) mothers with planned pregnancy	3 years after
Penfold (2007)<br><br>Ghana	Quasi-experimental with control groups and pretests<br><br>Pre and post intervention implementation cluster-sampled household survey (2002-2003, 2004-2005)	Delivery Fee Exemption Policy in 2003	FBD	2,922 women aged 15-58, of 2 regions, who delivered during the exemption fee period	▪ 2,922 women had 3,035 deliveries: 43% before the exemption and 57% after the intervention period<br>▪ Increased adjusted OR 1.83 [95%CI 1.44-2.32] (p<0.001) and 1.34 [95%CI 1.02-1.76] (p<0.05) of delivering in a health facility in Central and Volta regions, respectively; except women with no education in Volta region	1 year before and 1 year after
Byamugisha (2010)<br><br>Uganda	Quasi-experimental with control groups and pretests<br><br>Retrospective analysis of hospital records	Routine Opt-out HIV testing in ANC visits in 2006	HIV testing during ANC visits	54,429 ANC attendees and 469 male partners who were tested for HIV (2002-2009)	▪ From May 2002 to May 2006, 6,570/29,834 (22%) new ANC attendees tested for HIV vs 21,538/24,595 (87.6%) (2-tailed p=0.002) tested for HIV from June 2006 to 2009<br>▪ Before policy, 87.5% of men tested for HIV, and 100% of them after (2-tailed p=0.01)	4 years before and same year

	(2002-2006 and 2006-2009)				▪ 316/566 (55.8%) of HIV infected pregnant women used ARV for PMTCT before policy vs 855/1,147 (77.2%) after policy (2-tailed p=0.015)<br>▪ 172/566 (30.4%) of HIV infected pregnant women delivered in hospital before policy vs 464/1,147 (40.5%) after policy (2-tailed p=0.042)	
De Allegri (2011)<br><br>Burkina Faso	Cross-sectional<br><br>Three-stage cluster household survey (Feb-March 2009)	Abolition of ANC user fees, 2002<br><br>Hospital admission without pre-payment for all emergency cases, 2006<br><br>Subsidy for C-section, 2006<br><br>Subsidy for all deliveries 2007	ANC, DEL	435 women who reported a pregnancy 12 months prior to interview date (Feb-March 2009)	▪ OR of 4.3 among women with at least 3 ANC visits to deliver in health facility (p=0.001)<br>▪ OR of 28.42 among women living ≤ 5 km from health facility to delivery in facility (p=0.001)<br>▪ OR of 17.20 among Mossi pregnant women and OR of 8.32 among Peuhl women to deliver in facility (p=0.001)<br>▪ But lack of associations between age, parity, education, and household head characteristic and ANC seeking	2-3-7 years after
De Allegri (2012)<br><br>Burkina Faso	Quasi-experimental without control groups<br><br>Five repeated cross-sectional surveys (2006-2010)	Abolition of 80% user fees for FBD, 2006 (effective in January 2007)	FBD	1934 women who has completed a pregnancy 12 months before interview	▪ Increase of FBD from 49% in 2006 to 84% in 2010 (p<0.001)<br>▪ Concentration index >1, favouring women with higher SES, 2 years before policy implementation (p=0.05) and 3 years after (p<0.001)	1 year before and 0-1-2-3 years after
Dzakpasu (2012)<br><br>Ghana	Interrupted time-series<br><br>Time-series methods (DHS and cluster RCT) (2004-2009)	Free delivery care, 2005<br><br>Free National health Insurance for pregnant women, 2008	FBD (and health insurance coverage)	Out of 92,462 deliveries, 91,015 (98.4%) women with complete data (2004-2009)<br><br>27,841 (90%) who delivered (2008-2009) with complete insurance enrolment data	▪ Increase of 2.3% (p=0.015) and 7.5% (p<0.001) after the 2005 free delivery care and 2008 free NHIS policies (after adjusting for month of delivery and temporal trend)<br>▪ Concentration index for FBD of -0.0086 after the 2005 free care (period 1) and -0.0478 after the 2008 NHIS policies (period 2), favouring poorest women (p<0.001)<br>▪ Concentration index for FBD of 0.016 from period 1 to period 2, favouring richer women (p<0.001)<br>▪ Concentration index for FBD of -0.018 after the 2005 free delivery and after the 2008 NHIS policies, favouring poorer women (p<0.001)	1 before and 1 year after

El-Khouri (2012)<br><br>Mali	Quasi-experimental with control groups and no pretest<br><br>Nationally representative facility-based patient survey (of 16 public health sector centres and 9 hospitals that offer C-sections in 2010); comparing to DHS 2006)	Fee exemption policy for caesareans, 2005	C/S	2,477 women who had C-sections over 8-month period	▪ Richest 40% women with 1.67 times more to get a C-section in 2010 compared to those with a C-section in 2006 [95% CI 1.43-1.91]<br>▪ Poorest 40% women with 0.59 times more to get a C-section in 2010 compared to those with a C-section in 2006 [95% CI 0.47-0.71]<br>▪ Transport costs and road conditions as barriers to accessing C-section services in health facilities	5 years after
Bellows (2013)<br><br>Kenya	Quasi-experimental without control groups<br><br>Two cross-sectional household surveys (2004-2005 and 2006-2008)	Maternal Health Voucher Programme, 2006	FBD, SBA	4,362 women aged 12-54 years old<br><br>1,914 in 2004-2005, before the programme<br><br>2,448 in 2007-2008, after the programme	▪ Increased OR of 1.4 [95% CI 1.19-1.58; p<0.001] for FBD and OR of 1.2 [95% CI 1.02-1.36; p<0.01] for SBA after the programme implementation<br>▪ Variables associated with a statistically significant increased odds of having a FBD include: being aged 24-34 years old, having completed secondary education and being among the least poor<br>▪ The 'least poor' and women with ≥4 children, less likely to purchase voucher	2 years before and same year
Kengia (2013)<br><br>Tanzania	Quasi-experimental without control groups<br><br>Four cross-sectional surveys (DHS) (1992, 1996, 1999, 2004/5)	Health sector reform Plan of Action 1996-1999<br><br>Health sector reform Programme of Work 1999-2002	SBA	14,752 women aged 15-49 years old	▪ Overall % of SBA utilisation rate decreased from 1992-1999 and increased in 2004-2005<br>▪ Poorest and poor women's SBA utilisation decreased from 1992-1999, while that of middle income, rich and richest women increased<br>▪ From 1999-2004, it increased among poorest and poor women, and decreased among the other wealth quintile women	4 years before and 0-3 years after (first policy)<br><br>3-7 years before and 5 years after (second policy)
Obare (2013)<br><br>Kenya	Quasi-experimental with control groups and no pretest<br><br>Household survey comparing women exposed to the programme (since	Reproductive Health Vouchers, 2006-2011	ANC services, FP, DEL, PNC	2,527 women aged 15-49 years old<br>• Step 1: 2006-2008<br>• Step 2: 2008-2011	▪ OR of 1.5 [95% CI 1.0-2.1; p<0.05] among women exposed to the programme since 2006 to ever use family planning compared to those not exposed at all<br>▪ OR of 3.6 [95% CI 1.2-11.2; p<0.05] among Muslim/other/no religion to ever use family planning in past 12 months compared to those with Catholic religion	Same year

	2006) and those who are not (2006-2010)				▪ OR of 2.1 [95% CI 1.5-3.1; p<0.01] for FBD and OR of 2.0 [95% CI 1.4-2.8; p<0.01] for SBA among women who are exposed to the programme since 2006 compared to those not exposed at all<br>▪ No difference in ANC services among the women of the 2 groups<br>▪ Poor women significantly less likely to have delivered in a facility, have been assisted by SBA or received postnatal services compared to non-poor among women exposed to the programme since 2006, those not exposed in 2006-2010 and those not exposed at all<br>▪ Significantly higher proportion of poor women who have been exposed to the programme since 2006 compared with poor women who have not been exposed at all to the programme	
Skiles (2013)<br><br>Rwanda	Quasi-experimental with control groups and pretests<br><br>Cross-sectional survey (DHS): 2005: pre-intervention; 2007-2008: post intervention	Performance-based Financing, 2005	CTN, ANC, FBD	4,477 women aged 15-49 years old from intervention districts and 3,422 women in control districts	▪ In 2007, FBD improved significantly in all income groups (p<0.001) except for the poorest and the least poor<br>▪ In 2007, modern contraceptive use significantly increases in all income groups (with at p<0.01)<br>▪ No change over time in ANC≥4 among all income groups except for the middle-income group (p<0.01) and the less poor (p<0.05)	0-3 years after
Abrokwah, (2014)<br><br>Ghana	Quasi-experimental with control groups and no pretest<br><br>Cross-sectional survey (2005-2006)	Social Health Insurance, 2005	ANC	1,012 women aged 15-49 years old and who were pregnant	▪ Women who had access to insurance are more likely to seek (and spend at least 1 cent) on prenatal care compared to uninsured pregnant women (p=0.05 at baseline, and p=0.001 with interaction)<br>▪ Women who were in an area with health insurance actually spend less out-of-pocket on any spending compared to women in areas without health insurance	Same year
Fournier (2014)<br><br>Mali	Interrupted time series<br><br>Pre-intervention period of 30 months (January 1, 2003 to June 30, 2005)	Fee exemption for C-sections, 2005	C/S	5,375 women (of reproductive age)	▪ There has been an increase among all women living in villages with no healthcare facility, villages with healthcare facilities and in cities with a district hospital<br>▪ However, after policy implementation, there has been statistically significant (p<0.001) increase of 1% in C/section among women in villages with no	30 months before and up to 83 months after

	Post-intervention period of 83 months (July 1, 2005 to May 31<br>2012)				healthcare facility and of 5.7% increase among those living in cities with a district hospital, but not among those living in villages with a healthcare centre	
Frimpong (2014)<br><br>Ghana	Quasi-experimental without control groups<br><br>Retrospective cohort (January 2008 and August 2010)	National Health Insurance Scheme (2003)<br><br>Premium exemption for pregnant women, July 2008	ANC	1,411 women who conceived and delivered after the exemption policy	▪ Among women who conceived after the premium exemption, NHIS registration increased significantly to 71-73% among pregnant women in 2008-2009 and to more than 90% among women who delivered in 2009 ($p<0.01$)<br>▪ In hospitals and health centres, women registrants were more likely to receive extensive safe motherhood counseling during ANC vs those who are not registered<br>▪ In hospital and health centres, health insurance did not affect place of delivery<br>▪ Clients from CHC who are registered were significantly more likely to deliver at a health facility than others	5-7 years after
Ganle (2014)<br><br>Ghana	Cross-sectional<br><br>Retrospective cross-sectional Maternal Health Survey (2007)	User fee exemption for maternal healthcare policy, 2003-2005	ANC, DEL, PNC	10,370 women aged 15-49 years old	▪ ANC more common among women with live birth (98%) vs women with stillbirths (88%)<br>▪ The frequency of ANC higher among women with high school and higher education (7.9) vs women with no education (5.3), and among highest wealth quintile (8.3) vs lowest quintile (4.9)<br>▪ 88% of births of women with at least secondary education in health facility vs 31% of births of women with no education<br>▪ 92% women in the highest wealth quintile delivered institutionally vs 27% of women in lowest quintile<br>▪ More women living in urban areas delivered in health facilities vs women in rural areas	4 years after
McKinnon (2015)<br><br>Ghana<br>Senegal<br>Sierra Leone	Quasi-experimental with control groups and pretests<br><br>Representative household surveys (DHS) in 3 countries	User fee exemption for pregnant women<br><br>September 2003 in Ghana (for poorest regions first)	FBD	All women aged 15-49, between 2000 and 2012, totalling 150,541 live births	▪ Increased proportion of women delivering in health facilities across SES (wealth quartile, number of assets and maternal education) - But no difference in FBD increase after user fee removal among women in the poorest quartile and richest women	Pre-assessment for control countries and post-assessment for intervention countries with policy adoption

Control:<br>Cameroon<br>Congo (Brazzaville)<br>Ethiopia<br>Guinea<br>Mozambique<br>Tanzania	with user fee exemption implementation of policy and 3 control countries without policy implementation	January 2005 in Senegal (in most deprived provinces first)<br><br>April 2010 in Sierra Leone for all women and children under 5			▪ Little evidence that trends in the prevalence of facility delivery by any SES measures differed between the intervention and control countries<br>▪ Women in most materially deprived women with greater FDB prevalence, though not statistically significant<br>▪ Increased FBD prevalence among women with secondary education (p=004) vs those with no education	
Singh (2015)<br><br>Ghana	Mixed methods<br><br>Quantitative household and community leader surveys and qualitative interviews (May-June 2012)	NHIS in 2003-2005, with fee exemption for women and children under 3 months in 2008	ANC, FBD	969 women with children under 5	▪ Having insurance during the exact time of pregnancy was significantly associated with FBD (OR=2.5; CI: 1.3-4.5; p<0.01), but not with ANC visits<br>▪ Qualitative findings show a lack of understanding among women of who and what are covered<br>▪ Wealthier (X^2=17.0; df=4; p<0.002) and more educated women (X^2=40.3; df=4; p<0.000) more likely to have some insurance coverage<br>▪ Insurance did not address costs related to transport and extreme poverty experienced by some women	4-9 years after
Chama-Chiliba (2016)<br><br>Zambia	Quasi-experimental with control groups and pretests<br><br>Cross-sectional DHS (May 2002-September 2007)	Abolition of user fees in public health facilities, April 2006	FBD (in rural areas)	7,146 women aged 15-49, 5,410 children born between May 2002 and September 2007<br><br>1,500 women in 54 rural districts (fees abolished) in treatment group in specific region and 2,118 women in (fee paying) control regions	▪ Fee abolition with no significant effect on delivery location for home or public deliveries among women living in rural areas – But significant positive effect on deliveries in private or faith-based health facilities for rural women<br>▪ No significant difference in ANC between treatment and control groups<br>▪ Factors: distance and drugs availability in health facilities	4 years before and 1 year after
Johnson (2016)<br><br>Ghana	Quasi-experimental without control groups	Cash and carry policy, before 1998<br><br>Free ANC policy (1998-2003)	SBA	Thousands of women giving birth, depending on each wave of DHS	▪ Overall increased percentage of SBA over different periods of policy implementation among different sub-groups (p<0.01): women with no formal education and those with secondary/higher education; no formal education, primary education	5 years before and 3 years after

	Four rounds of DHS (1993, 1998, 2003, 2008)	Free delivery care policy (2003-2005) and abolished in 2007<br><br>NHIS in 2005, with integration of exemption fees for pregnant women in 2008			and secondary/higher education among women's partner; women from all wealth quintiles; and women in urban and rural residence<br>▪ But: Northern and Southern district divide persist, maybe explained by the North being poorer and with women with low education (less aware of the policies?), as SES variables do not explain it all	
Langlois (2016)<br><br>Burkina Faso	Quasi-experimental without control groups<br><br>Quasi-experimental (2008 and 2010)	National policy to subsidize SBA, 2006-2007	SBA	1,260 women aged 15-49 (pre-subsidy) and 1,395 women aged 15-49 (post subsidy)<br><br>1st survey in 2008<br>2nd survey in 2010	▪ Increase in adjusted rate of SBA among all SES strata of women, strongest among lowest SES women with increased rate of SBA sustained over time: 45% (95% CI=19-77%) immediately upon subsidy policy introduction; 46% (95% CI=20-78%) higher at 6 months; 47% (95% CI=20-78%) higher at 1 year; and 48% (95% CI=21-81%) higher 2 years after policy introduction<br>▪ Apparent decline in SES inequities in accessible obstetric care in Burkina Faso?	2-4 years after
Leone (2016)<br><br>Ghana<br>Burkina Faso<br><br>Control:<br>Cameroon<br>Nigeria<br>Zambia	Mixed methods<br><br>Quasi-experimental intervention countries, with last survey conducted at least 3 years after policy: Burkina Faso (circa 2010) and Ghana (circa 2006)<br><br>Control countries: Cameroon, Zambia and Nigeria<br><br>Key informant interviews	Burkina Faso: 80% reduction in user fees, 2007<br><br>Ghana: Institutional birth fee exemption, 2003-2005<br><br>No user fee policy in control countries	C/S, FBD	187,310 women	▪ Increase FBD from 40% to 70% and from 45% to 60% in Burkina Faso and Ghana, respectively<br>▪ In Burkina Faso and Ghana, women from no to higher education more likely to have FBD, whereas those with no and primary education were more likely to have C/section (p=0.000 and p=0.017); women living rural and urban areas more likely to have FBD, whereas it is only those living in urban areas more likely to have C/section (p=0.000)<br>▪ Compared to poorest women in all 5 countries, women who are of average wealth, rich and richest are more likely to have C/sections (p=0.000)	At least 3 years after

Manthalu (2016)<br><br>Malawi	Quasi-experimental with control groups and pretests<br><br>Yearly panel data (2003-2010)	Exemption fees for health care policy, with mission health facilities, 2006	ANC, DEL, PNC	Women receiving maternal health services from 142 mission health facilities which signed service level agreements (SLA)	▪ Significant difference between women receiving services from SLA and no SLA health facilities in at least 1 ANC during pregnancy and FBD ($p<0.01$), but no difference in 1ANC during 1st trimester and postpartum care visits	3 years before and 4 years after
Vallières (2016)<br><br>Sierra Leone	Quasi-experimental without control groups<br><br>Cross-sectional survey (October-November 2011)	Free Health Care Initiative (FHCI) for pregnant and lactating mothers, 2010	FP, ANC, SBA FBD, PMTCT, Child health	205 pregnant women from villages in the riverine area, and 136 pregnant women from villages in the mainland area	▪ Significant difference in SBA % post-FHCI among riverine (44.3%) and mainland (64.9%) women ($p=0.000$)<br>▪ Significant difference in TBA in post-FHCI in riverine (55.7%) and mainland (33.9%) women ($p=0.000$), whereas there was no significant difference in pre-FHCI (50.8% in riverine women and 44.4% in mainland women)<br>▪ Significant difference in FBD % in post-FHCI among riverine (41.3%) and mainland (61.2%) women ($p=0.000$)<br>▪ Significant difference in FP % in post-FHCI among riverine (28.4%) and mainland (44.8%) women ($p=0.000$)<br>▪ No difference in PMTCT in women in the riverine and mainland region<br>▪ Factors related to transport and more difficult terrain in the riverine area, as well as poor working and living conditions of health workers (worse in remote areas like in the riverine areas?)	1 year after
Ajayi (2017)<br><br>Nigeria	Quasi-experimental without control groups<br><br>Cross-sectional surveys (DHS 2008, 2013) and survey (2016)	National free maternal and child healthcare programme, 2012	ANC, FBD, SBA	1,227 women aged 15-49 in 3 states	▪ 33.6% of all women benefited from free maternal health services, those from middle SES (38.3%, $p<0.001$) and who lived in a community where a health facility was available (37.2%, $p<0.001$) benefited most<br>▪ No clear trends in ANC and FBD from 2008, 2013 and 2016 in 3 states<br>▪ Increase in SBA in 3 states from 2013 to 2016	4 years before and 1-4 years after
Wang (2017)<br><br>Ghana<br>Rwanda<br>Indonesia	Quasi-experimental with control groups and no pretest	NHIS: 2003 in Ghana, 2004 in Rwanda	ANC1, ANC4, ANC during 1st trimester, FBD	Women aged 15-49 who are insured and those not insured (matched)	▪ 40.1% in Ghana and 73.1% in Rwanda of total women covered by health insurance<br>▪ Health insurance coverage positively associated with employment and richest households in	5-6 years after

N.B. Only data from sub-Saharan Africa reported here	DHS in Ghana (2008) and Rwanda (2010)				Ghana, and unemployment and poorest household in Rwanda<br>▪ Before matching, significant difference between uninsured and insured women in terms of ANC1, but not after matching in Ghana. Significant difference of ANC1 before and among matching between both groups in Rwanda, favouring those with insurance<br>▪ Before matching, ANC in the 1st trimester positively associated with health insurance coverage in Ghana and Rwanda, but not after matching<br>▪ Significant differences before and after matching for FBD between insured and not insured in Ghana and Rwanda	
Calhoun (2018)<br><br>Kenya	Quasi-experimental without control groups<br><br>Longitudinal survey, baseline (2010) and endline (2014)	Removal of delivery fees in public facilities policy, 2013	FBD in public facilities	2,793 women at baseline and 1,232 women at endline (15-49 years old, unweighted)	▪ Women more significantly likely to deliver in a public health facility than at home or a private facility post policy<br>▪ Among the poor women, increase in public health sector delivery (from 40 to 46%) and in private sector (from 36 to 45%), and decrease in home delivery (from 24 to 10%)	3 years before and 1 year after
Dennis (2018)<br><br>Kenya	Quasi-experimental with control groups and pretests<br><br>Repeated cross-sectional in 4 intervention and 3 comparison counties, looking at three periods: 2005-2009, 2010-2013 and 2013-2016	Maternal health voucher programme (2006-2016), Free Maternal Services Policy, 2013	ANC4+, FBD, PNC, complete care	5,323 women aged 15-49 who gave birth or was pregnant in the past 12 months	▪ ANC4+ increased for women living in both voucher and comparison counties in pre and post-policy implementation period; OR 1.46 among women in voucher counties to have ANC4+ vs comparison during free maternal service period (p=0.006)<br>▪ No difference in FBD during pre and post-policy combined with voucher programme period, but OR 1.65 among women in voucher counties to have FBD vs comparison, following the post-policy implementation period (p=0.008)<br>▪ OR 1.73 among women in voucher counties to have PNC vs comparison following the post-policy implementation period (p=0.001)<br>▪ In all periods, more than 60% of maternal services utilised in the public sector (vs private), with a decline of service utilisation in private sector	1 year before and 4-7 years after

					during post-policy period combined with free maternal service period	
Obare (2018)<br><br>Kenya	Quasi-experimental: interrupted time-series<br><br>Repeated cross-sectional survey (DHS): 2003, 2008-9, 2014	User fee policy shifts: 2004 "10/20 policy", 2007 "user fee removal of 10/20 policy in public facilities" and 2013 "free maternity services"	ANC, FBD	8,195, 8,444 and 31,079 women who had ever given birth in 2003, 2008-9 and 2014 respectively	▪ ANC and FBD steadily increased between 2003 and 2014<br>▪ Women with ANC from public health facilities increased from 71% in 2003 to 83% in 2008-9, while services from private facilities decreased over the same period<br>▪ No significant changes in public FBD following the 2004 10/20 and 2014 free maternity policies among poor or rural women<br>▪ After the 2004 10/20 policy, there was a statistically significant increase in home deliveries among all women<br>▪ A statistically significant increase in public FBD among richer women, accompanied by a decrease in home deliveries after 2007 policy	1 year before 1st policy and 1 year after for last 2nd and 3rd policies
Ogundele (2018)<br><br>Ghana<br>Nigeria	Quasi-experimental without control groups<br><br>Three series of cross-sectional surveys (DHS): in 2003, 2008 and 2014 in Ghana, and in 2003, 2008 and 2013 in Nigeria	Ghana fee exemption for maternity care policy, 2005 (initiated in 2003)<br><br>Nigeria NHIS, 2005 (initiated in 1999)	FP, ANC and delivery	Women aged 15-49 who gave at least one birth in the 5 years prior to survey, in both countries	▪ Regarding SRH service utilisation examined, there is a decline of inequality between richer and poorer in Ghana, while it is non-pro-poor in Nigeria<br>▪ The use of FP information is significantly favouring poorer women in Ghana and pro-rich in Nigeria ($p \leq 0.01$)<br>▪ The use of ANC services at government health facility is pro-poor in both countries ($p \leq 0.01$), while delivery at home is also mostly used by poorer women in both countries ($p \leq 0.01$)<br>▪ C/S is pro-rich in both countries ($p \leq 0.01$)	Ghana: 0-5-11 years after<br><br>Nigeria: 4-9-14 years after
Ravit (2018)<br><br>Benin<br>Mali	Quasi-experimental without control groups<br><br>Repeated cross-sectional surveys (DHS): in Benin in 2001, 2006 and 2011-12, and in Mali in 2001, 2006 and 2012-13	User fee policy exemption for C/S in selected public and private health facilities in Benin, 2009, and user fee removal reform, including C/S in Mali, 2005	C/S	23,266 and 24,036 women aged 15-49 who delivered a live-born child in the past 5 years prior to interview, in Benin and Mali, respectively	▪ In Benin, no significant difference in C/S between urban and rural or educated and non-educated women; there is a difference favouring richer women vs poorer women having access to C/S ($p \leq 0.001$), and no difference between rich/poor before and after policy adoption<br>▪ In Mali, significant difference between educated and non-educated in accessing C/section before and after policy implementation ($p = 0.043$); no difference between rich and poor between before and after policy implementation	1-8 years before and 2-8 years after

Ravit (2018)<br><br>Benin<br>Mali<br><br>Control:<br>Cameroon<br>Nigeria	Quasi-experimental with control groups and pretests<br><br>Repeated cross-sectional surveys (DHS):<br><br>Intervention countries:<br>Benin: 1996, 2001, 2006, 2011-12<br>Mali: 1995-96, 2001, 2006, 2012-13<br><br>Control countries:<br>Cameroon: 1991, 1998, 2004, 2011<br>Nigeria: 1990, 2003, 2008, 2013	User fee policy exemption for C/S in selected public and private health facilities in Benin, 2009, and user fee removal reform, including C/S in Mali, 2005	C/S and FBD	46,362 women who delivered a live child in the last 3 years before the interview in the policy group, and 53,438 women in the non-policy group	▪ Adjusted OR=1.36 (95%CI 1.11 to 1.66; ($p \leq 0.01$) to have access to C/S; adjusted OR=2.71 (95%CI 1.70 to 4.32; ($p \leq 0.001$) among non-educated women; adjusted OR=2.02 (95%CI 1.48 to 2.76; ($p \leq 0.001$) among women living in rural areas; and adjusted OR=3.88 (95%CI 1.77 to 4.72; ($p \leq 0.001$) among middle-class wealth index<br>▪ The policy contributes to adjusted OR=1.68 (95%CI 1.48 to 1.89; ($p \leq 0.001$) in FBD	3-13 years before and 2-8 years after

Legend:

ANC: Antenatal check-up
CTN: Contraception
C/S: Caesarian section
DEL: Delivery

FBD: Facility-Based Delivery
FP: Family Planning
NHIS: National Health Insurance Scheme
PMTCT: Prevention of Mother to Child Transmission

PNC: Postnatal Care
SBA: Skilled Birth Attendants

Table 2 SRH service utilisation results by quasi-experimental design and significance of results

	Positive results[1]				Mixed results[2]				No significant results[3]				Total
Quasi-experimental design	A	B	C	D	A	B	C	D	A	B	C	D	
Abortion services													-
Family planning / contraception	**1**<br>Ogundele (2018)	**1**<br>Obare (2013)	**1**<br>Skiles (2013)										**3**
Antenatal care	**1**<br>Ogundele (2018)	**2**<br>Abrokwah (2014)<br>Wang (2017)					**2**<br>Skiles (2013)<br>Manthalu (2016)		**2**<br>Frimpong (2014)<br>Ajayi (2017)	**1**<br>Obare (2013)		**1**<br>Obare (2018)	**9**
Facility-based delivery	**3**<br>De Allegri (2012)<br>Bellows (2013)<br>Vallières (2016)	**2**<br>Obare (2013)<br>Wang (2017)	**5**<br>Penfold (2007)<br>Skiles (2013)<br>Leone (2016)<br>Manthalu (2016)<br>Ravit (West Africa, 2018)	**1**<br>Dzakpasu (2012)	**1**<br>Frimpong (2014)		**2**<br>Chama-Chiliba (2016)<br>Dennis (2018)	**1**<br>Obare (2018)	**2**<br>Ajayi (2017)<br>Calhoun (2018)		**2**<br>McKinnon (2015)<br>Leone (2016)		**19**
Skilled birth attendants	**4**<br>Bellows (2013)<br>Johnson (2016)<br>Langlois (2016)<br>Vallières (2016)				**1**<br>Kengia (2013)				**1**<br>Ajayi (2017)				**6**
Caesarian section	**1**<br>Ogundele (2018)	**1**<br>El-Khouri (2012)	**2**<br>Leone (2016)<br>Ravit (West Africa, 2018)	**1**<br>Fournier (2014)	**1**<br>Ravit (Benin, Mali, 2018)								**6**

Postnatal care			**1**<br>Dennis (2018)										**1**
HIV testing during ANC			**1**<br>Byamugisha (2010)						**1**<br>Vallières (2016)				**2**
Total	**10**	**6**	**10**	**2**	**3**	**-**	**4**	**1**	**6**	**1**	**2**	**1**	**46[a]**
Total	**28**				**8**				**10**				**46[a]**

[a] 27 studies analysed multiple SRH results (including one of the mixed methods studies which used a quasi-experimental design for its quantitative component).

Notes:
[1] Positive results: If all results are reported to be statistically significant and improved outcomes.
[2] Mixed results: A mixed of positive, negative, statistically significant and not statistically significant outcomes.
[3] Negative results: Not statistically significant or significance not reported.

Legend:
A: Quasi-experimental designs without control groups
B: Quasi-experimental designs that use control groups but no pretest
C: Quasi-experimental designs that use control groups and pretests
D: Interrupted time-series designs
